# From inflammatory phenotypes to clinical decisions: a stratified diagnostic and therapeutic framework for CIP

**DOI:** 10.3389/fimmu.2026.1961402

**Published:** 2026-09-22

**Authors:** Shanshan Lin, Wenye Huang, Binlong Zhang, Xiaomin Yang, Haocheng Zhao

**Affiliations:** 1 Department of Respiratory Medicine, Yueqing People’s Hospital, Yueqing, Zhejiang, China; 2 Department of Hematology, Yueqing People’s Hospital, Yueqing, Zhejiang, China; 3 Department of Gastroenterology, Yueqing People’s Hospital, Yueqing, Zhejiang, China

**Keywords:** CIP, immune checkpoint inhibitor, immune-related adverse events, inflammatory phenotype, phenotype-guided management, risk stratification, steroid-refractory pneumonitis

## Abstract

**Background:**

Checkpoint inhibitor pneumonitis (CIP) is an uncommon but potentially life-threatening immune-related adverse event that may interrupt effective anticancer therapy and require prolonged immunosuppression. Its clinical presentation, radiographic appearance, inflammatory biology, and response to corticosteroids vary substantially, while infection, radiation pneumonitis, tumor progression, pulmonary embolism, and pre-existing interstitial lung disease may produce overlapping findings. Conventional severity grading remains important for communication and triage but does not fully capture this heterogeneity.

**Review content:**

This narrative review synthesizes clinical, radiographic, immunological, and treatment-response evidence to develop a framework for phenotype-guided CIP management. We conceptualize CIP as a dynamic pulmonary immune syndrome and organize its assessment across four dimensions: clinical severity, radiographic phenotype, inflammatory and immune phenotype, and corticosteroid responsiveness. Emerging evidence involving CD4+ and CD8+ memory and tissue-resident T cells, Th17.1 and Tfh-like programs, pro-inflammatory monocytes and macrophages, cytokine networks, and humoral responses is linked to clinically relevant questions while remaining hypothesis-generating. Three patterns are emphasized: low-inflammatory-burden, steroid-responsive disease; high-risk progressive disease; and persistent immune-activation or steroid-refractory disease. The framework connects these patterns with diagnostic reassessment, multidisciplinary evaluation, corticosteroid treatment, recognition of treatment failure, immunomodulatory escalation, chronic pulmonary follow-up, and individualized immune checkpoint inhibitor rechallenge.

**Conclusions:**

CIP should not be managed as a single toxicity defined by grade alone. Joint interpretation of clinical trajectory, imaging, inflammatory signals, and corticosteroid response may improve risk stratification and support more proportionate diagnostic and therapeutic decisions. This framework is a clinically grounded heuristic rather than a validated algorithm and requires prospective multicenter evaluation.

## 1 Introduction

Immune checkpoint inhibitors (ICIs), particularly agents targeting PD-1, PD-L1, and CTLA-4, have reshaped the treatment landscape of lung cancer and several other solid tumors (1–3). Their clinical value is no longer confined to a single disease setting; rather, they are now incorporated across metastatic, perioperative, and selected adjuvant strategies (4, 5). This expansion has been accompanied by a parallel rise in immune-related adverse events, a group of toxicities that can affect almost any organ system and frequently require treatment interruption, corticosteroid exposure, or multidisciplinary management (6–8). Among these toxicities, Checkpoint inhibitor pneumonitis (CIP) occupies a distinctive position because it may be uncommon yet clinically consequential, with the potential to cause rapid respiratory deterioration, prolonged immunosuppression, and interruption of otherwise effective anticancer therapy (9–11).

CIP is particularly challenging because it does not present as a single, uniform clinicopathologic entity (9, 12, 13). Some patients develop incidentally detected ground-glass opacities with minimal symptoms, whereas others present with hypoxemic respiratory failure and diffuse parenchymal injury (13–15). The clinical course may be abrupt or indolent, self-limited or relapsing, steroid responsive or steroid refractory (16–18). These differences are not merely semantic. They influence the urgency of diagnostic work-up, the threshold for hospitalization, the pace of corticosteroid escalation, and the safety of later immune checkpoint inhibitor rechallenge (19–21). In daily practice, clinicians are often required to make these decisions before the biological nature of the lung injury is fully understood (22, 23).

The current diagnostic and therapeutic approach to CIP remains largely anchored to severity grading systems that were designed to standardize adverse event reporting rather than to capture biological heterogeneity (19, 24). CTCAE grading is useful for communication and triage, but it does not fully distinguish inflammatory burden, radiographic extent, dominant immune-cell programs, or the likelihood of corticosteroid response (10, 17, 22, 25).This limitation matters because patients with the same grade may differ substantially in trajectory and management needs (11, 14, 15). A mild-appearing radiographic phenotype may still reflect active immune injury, whereas some severe presentations may already have evolved into diffuse alveolar damage or steroid-resistant inflammation (13, 17, 26). At the same time, the differential diagnosis of CIP is broad and clinically consequential, including infection, radiation pneumonitis, tumor progression, pulmonary embolism, and exacerbation of pre-existing interstitial lung disease (12, 27, 28).

Over the past several years, advances in bronchoalveolar lavage, tissue-based profiling, single-cell transcriptomics, and real-world cohort studies have begun to reveal that CIP is immunologically heterogeneous (10, 25, 29, 30). Distinct patterns of T-cell activation, myeloid-cell participation, cytokine signaling, and humoral immune responses have been described, but these observations have not yet been translated into a practical framework for bedside decision-making (10, 31, 32). In parallel, emerging data suggest that corticosteroid responsiveness itself may represent an informative clinical axis, helping to separate low-inflammatory-burden cases from progressive or steroid-refractory disease (17, 18, 33). These developments support a more integrated view of CIP, one that links clinical severity, radiographic phenotype, immune-inflammatory profile, and treatment response (**Table 1**) (22, 34, 35).

**Table 1:** Phenotype-guided clinical summary of CIP.

Phenotype	Bedside profile	Immune/radiographic cues	Practical implication
Incidental/low-burden	Incidental CT finding; minimal symptoms; oxygenation preserved.	Focal GGO or OP-like opacity; low inflammatory burden.	Observe or hold ICI; repeat imaging if the pattern evolves.
Early inflammatory/mildly symptomatic	Cough, dyspnea, or low fever; modest O2 need; uncertain course.	Limited OP/NSIP/HP-like change; evolving inflammatory signal.	Hold ICI; consider steroids; reassess in 48–72 h.
Hypoxemic progressive	Desaturation, rising O2 need, or rapid clinical decline.	Bilateral GGO/consolidation; expanding burden on serial CT.	Admit; steroids; exclude infection, PE, and progression.
Severe diffuse injury	Respiratory failure; high-flow oxygen or ventilatory support.	Diffuse bilateral opacities; DAD/ARDS-like pattern.	IV steroids, ICU support, and early multidisciplinary review.
Steroid-refractory/relapsing	No early response or relapse on taper; persistent symptoms.	Non-resolving opacities; serial imaging remains abnormal.	Re-check diagnosis; consider phenotype-informed escalation.
Chronic/fibrotic/rechallenge	Residual dyspnea, PFT decline, or recurrent symptoms.	Residual interstitial change or fibrotic residue.	Prolonged taper and follow-up; selective rechallenge after recovery.

ARDS, acute respiratory distress syndrome; CIP, checkpoint inhibitor pneumonitis; CT, computed tomography; DAD, diffuse alveolar damage; GGO, ground-glass opacity; HP, hypersensitivity pneumonitis; ICI, immune checkpoint inhibitor; ICU, intensive care unit; NSIP, nonspecific interstitial pneumonia; OP, organizing pneumonia; O2, oxygen; PE, pulmonary embolism; PFT, pulmonary function test.

These categories are a clinical heuristic rather than a validated classification system and may overlap or evolve over time.

In this narrative review, we propose a stratified diagnostic and therapeutic framework for CIP that is intended to be clinically usable rather than conceptually exhaustive. Our central premise is that CIP is best understood as a dynamic pulmonary immune syndrome with multiple overlapping phenotypes, not as a single toxic event defined only by grade (18, 25, 29). We therefore organize the discussion around four dimensions: clinical severity, radiographic phenotype, inflammatory and immune phenotype, and corticosteroid responsiveness. This framework is designed to support a more nuanced approach to diagnosis, risk stratification, treatment escalation, and immune checkpoint inhibitor rechallenge, while remaining grounded in observations that are accessible to practicing clinicians (19, 36, 37). Detailed discussion of the framework is provided in the later phenotype-based sections.

The novelty of this framework does not lie in proposing entirely new individual variables. Clinical severity, radiographic findings, inflammatory markers, immune-cell features, and treatment response have each been discussed in previous guidelines and reviews. Rather, the framework explicitly integrates these observations into a single clinically oriented model. Clinical severity, radiographic phenotype, and inflammatory/immune phenotype describe current risk, structural burden, and biological activity, whereas corticosteroid responsiveness is incorporated longitudinally as a treatment-response modifier. The framework is therefore intended to complement, rather than replace, CTCAE grading by connecting baseline assessment with clinical trajectory and treatment behavior.

## 2 Scope and evidence base

This narrative review focuses on the clinical and translational evidence most relevant to CIP. It emphasizes literature published between 2019 and 2026, while retaining earlier landmark studies when they help define key clinical patterns, imaging phenotypes, immune mechanisms, or management principles. Rather than attempting exhaustive coverage, we selected evidence that can inform bedside reasoning, particularly when diagnostic uncertainty, corticosteroid responsiveness, or immune checkpoint inhibitor rechallenge is being considered.

The evidence base includes high-impact reviews, clinical cohorts, real-world studies, systematic reviews and meta-analyses, clinical practice guidelines and consensus statements, and mechanistic studies using bronchoalveolar lavage, tissue profiling, cytokine analysis, single-cell approaches, or T-cell receptor sequencing. This scope was chosen to support a clinically oriented synthesis of phenotypic heterogeneity and treatment decision-making rather than a formal systematic review of all available studies.

## 3 Clinical landscape and heterogeneity of CIP

### 3.1 Epidemiology

CIP is generally less frequent than many dermatologic, endocrine, or gastrointestinal immune-related adverse events, but its clinical weight is disproportionate to its incidence (6, 9, 38). Reported rates vary across clinical trials, real-world cohorts, tumor types, treatment regimens, and diagnostic definitions (11, 39, 40). Trial populations often exclude patients with poor pulmonary reserve, active interstitial lung disease, uncontrolled infection, or complex thoracic treatment histories; therefore, trial-based estimates may underrepresent the burden encountered in routine oncology practice (11, 14, 22). In real-world settings, the threshold for imaging, the intensity of surveillance, and the willingness to classify ambiguous pulmonary infiltrates as CIP may further influence reported incidence (11, 12, 23).

Representative estimates illustrate the range of reported incidence. In a systematic review and meta-analysis of 20 PD-1 inhibitor monotherapy trials, the overall incidence of pneumonitis was 2.7% (95% CI, 1.9%-3.6%), whereas the incidence of grade 3 or higher pneumonitis was 0.8% (95% CI, 0.4%-1.2%) (39). The incidence was higher in patients with NSCLC than in patients with melanoma, for both all-grade pneumonitis (4.1% vs 1.6%) and grade 3 or higher pneumonitis (1.8% vs 0.2%). In melanoma trials, combination therapy was associated with a higher incidence than monotherapy for both all-grade pneumonitis (6.6% vs 1.6%) and grade 3 or higher pneumonitis (1.5% vs 0.2%) (39). In a real-world cohort of patients with lung cancer treated with immune checkpoint inhibitors, the incidence of CIP was 9.5%, and 27% of affected patients died while CIP remained ongoing (11). These findings should be interpreted in the context of differences in patient selection, tumor type, treatment regimen, surveillance intensity, and diagnostic criteria rather than as directly comparable estimates.

Patients with lung cancer represent a particularly important population for CIP research and clinical decision-making (11, 12, 41). Several factors may converge in this setting: prior smoking exposure, chronic obstructive pulmonary disease, emphysema, interstitial abnormalities, previous thoracic radiotherapy, tumor-related airway or parenchymal changes, and repeated exposure to cytotoxic agents or targeted therapies (14, 42, 43). These overlapping conditions complicate both risk attribution and diagnosis (12, 13). As a result, CIP in lung cancer is rarely a purely immunological problem considered in isolation; it usually emerges within a pre-existing pulmonary and oncologic context (22, 44).

### 3.2 Clinical severity spectrum

The clinical spectrum of CIP ranges from asymptomatic radiographic abnormalities to life-threatening respiratory failure (9, 13, 24). At one end, patients may have limited ground-glass opacities or organizing pneumonia-like changes detected on surveillance imaging, without cough, dyspnea, fever, or oxygen desaturation (13, 45). These cases may be managed with close monitoring, temporary treatment interruption, or low-intensity intervention depending on trajectory and oncologic context (19–21). At the other end, patients may present with rapidly progressive dyspnea, diffuse bilateral infiltrates, hypoxemia, and a clinical picture resembling acute lung injury or acute respiratory distress syndrome (17, 26, 46).

Between these extremes lies the group most frequently encountered in clinical practice: symptomatic but initially non-critical pneumonitis (9, 11, 44). These patients may have cough, exertional dyspnea, low-grade fever, chest tightness, or a new oxygen requirement, yet their future trajectory is often uncertain at presentation (12, 33). Some improve promptly after corticosteroid initiation, whereas others deteriorate despite apparently appropriate management (17, 33). This variability is one reason why a single severity grade at diagnosis may be insufficient (18, 22). The same initial grade can conceal different radiographic burdens, inflammatory states, pulmonary reserve, and probabilities of steroid responsiveness (14, 15, 17).

### 3.3 Risk factors

Risk factors for CIP should be interpreted as cumulative and context dependent rather than as isolated predictors (47–49). Pre-existing interstitial lung disease is one of the most clinically relevant risk factors because it may reduce pulmonary reserve, complicate radiographic interpretation, and increase the risk of severe or irreversible lung injury (14, 42, 43). Chronic obstructive pulmonary disease, emphysema, and smoking history may also increase vulnerability, although their effects can be difficult to separate from lung cancer biology and treatment exposure (11, 41, 48). Prior thoracic radiotherapy is another important modifier, both because it may create a region of susceptible lung tissue and because radiation pneumonitis can closely resemble CIP on imaging and symptoms (12, 50).

Treatment-related factors also contribute to heterogeneity (38, 40, 51). Combination immunotherapy, particularly regimens involving CTLA-4 blockade together with PD-1 or PD-L1 inhibition, tends to produce broader immune activation and may increase pulmonary toxicity risk in susceptible patients (39, 51, 52). Chemoimmunotherapy and perioperative immunotherapy have introduced new clinical scenarios in which lung injury may reflect overlapping contributions from chemotherapy, surgery, radiation, infection, tumor response, or immune activation (4, 5, 53). In these settings, the question is often not whether a patient has pure CIP, but whether immune-mediated pneumonitis is sufficiently likely and clinically important to guide management (19, 28, 54).

Laboratory and clinical markers such as neutrophil burden, inflammatory indices, albumin level, baseline oxygenation, and early treatment response may help refine risk assessment, but none currently functions as a definitive diagnostic or prognostic biomarker (17, 33, 44). Their value is greatest when interpreted alongside symptoms, imaging, prior lung disease, treatment timing, and microbiological evaluation (19, 28, 55). This integrated interpretation is particularly important because overdiagnosis may lead to unnecessary corticosteroids and interruption of effective cancer therapy, whereas underdiagnosis may delay treatment of a potentially fatal pulmonary toxicity (12, 23, 55).

### 3.4 Dynamic clinical course

CIP should be viewed as a dynamic process rather than a static event (16–18). A useful clinical model includes several overlapping phases: initial onset, inflammatory progression, early corticosteroid response, steroid-refractory or steroid-relapsing disease, chronic or fibrotic evolution, and post-resolution assessment for possible ICI rechallenge (18, 36, 37). Not every patient passes through each phase, but this temporal framework reflects how clinicians actually encounter the disease (19, 23). A patient who appears stable on day one may worsen over the next several days, while another with initially extensive radiographic disease may improve rapidly once immunosuppression is started (17, 33).

The dynamic nature of CIP has practical implications. First, diagnosis is often provisional and must be revisited as microbiological results, serial imaging, oxygenation trends, and steroid response become available (12, 28, 55). Second, treatment intensity should be guided not only by baseline grade but also by trajectory (19–21). Third, corticosteroid responsiveness is itself clinically informative (17, 18, 33). Early improvement may support continued tapering and monitoring, whereas persistent hypoxemia, radiographic progression, or relapse during tapering should prompt reconsideration of alternative diagnoses, occult infection, insufficient immunosuppression, or a steroid-refractory inflammatory phenotype (16, 17, 35).

This clinical heterogeneity provides the rationale for moving beyond a one-dimensional classification of CIP (22, 34, 35). Incidence, severity, risk factors, and clinical course are all important, but none alone adequately explains why some patients recover quickly, others require prolonged immunosuppression, and a smaller subset develops progressive or recurrent lung injury (9, 11, 17). A more useful framework should therefore integrate the clinical context with radiographic pattern, inflammatory phenotype, and treatment response (18, 23, 54). These dimensions form the bridge between the observed clinical landscape and the phenotype-guided approach proposed in this review.

## 4 Inflammatory and immunological phenotypes of CIP

The clinical heterogeneity of CIP is increasingly supported by evidence that the pulmonary immune response is not uniform across patients (10, 29, 30). Although the available mechanistic literature remains limited by small sample sizes, tissue accessibility, and observational design, studies using bronchoalveolar lavage fluid, lung tissue, cytokine profiling, T-cell receptor sequencing, and single-cell transcriptomics suggest that CIP involves interacting T-cell, myeloid-cell, cytokine, and humoral immune programs (22, 31, 56). These findings should not be interpreted as definitive causal pathways in every patient (22, 35). Rather, they provide a biological vocabulary for understanding why some cases are mild and steroid responsive, whereas others are progressive, relapsing, or refractory (17, 18, 33).

### 4.1 T-cell-dominant inflammation

T-cell activation is central to the conceptual framework of CIP. Inhibition of PD-1, PD-L1, or CTLA-4 can restore antitumor immunity, but the same process may also lower the threshold for immune injury in normal tissues. In the lung, this may occur in the setting of pre-existing inflammation, shared tumor-lung antigenic signals, prior tissue damage, or impaired local immune regulation (10, 57, 58). The resulting pulmonary inflammation is not merely an excess of lymphocytes; rather, it appears to include distinct T-cell subsets with different migratory, effector, memory, and regulatory properties (10, 30, 59).

One repeatedly observed feature is enrichment of memory-like CD4+ T-cell populations in bronchoalveolar compartments (10, 59, 60). CD4+ central memory T cells may have enhanced capacity for persistence, trafficking, and rapid reactivation, which could help explain why some patients experience prolonged inflammation or relapse during corticosteroid tapering (16, 18, 61). However, their presence should be interpreted as a marker of an activated immune environment rather than proof that they alone drive lung injury. Clinically, this phenotype may be relevant because persistent memory T-cell activation could contribute to delayed resolution even after the initial inflammatory trigger has been suppressed (16, 17, 62).

Th17.1-like cells represent another important inflammatory population (29, 63). These cells express features associated with both Th1 and Th17 biology, including IFN-gamma, IL-17A, and GM-CSF-related programs (29, 64, 65). Their potential interaction with IL-23-producing myeloid cells suggests a self-amplifying inflammatory circuit in which T-helper activation and monocyte recruitment reinforce each other (30). This axis is particularly relevant to the concept of steroid-refractory CIP because Th17-skewed inflammation in other immune-mediated diseases may show variable corticosteroid sensitivity (63, 64). In CIP, this remains a hypothesis-generating but clinically attractive explanation for persistent inflammation, especially when conventional corticosteroid therapy fails to produce early improvement (29, 35).

CD8+ effector memory and tissue-resident memory T cells may be especially important in local tissue injury (57, 66). These cells are positioned to persist within lung tissue and respond rapidly to antigenic stimulation (58, 60, 62). Increased cytotoxic signatures, tissue localization, and clonal expansion have been described in some studies, raising the possibility that local CD8+ T-cell responses contribute to epithelial or alveolar damage (57, 60, 66). The concept of tumor-lung antigenic overlap is biologically plausible, but it should be framed cautiously; current evidence suggests association rather than universal causation (22, 58). From a clinical perspective, CD8+ tissue-resident memory activation may help explain localized recurrence, persistent radiographic abnormalities, or rapid reactivation after immune rechallenge in selected patients (16, 36, 37).

Tfh-like cells and regulatory T cells add further complexity. Enrichment of Tfh-like populations may indicate antigen-driven immune activation and may provide a link between T-cell responses and B-cell or antibody-mediated features. In contrast, impaired regulatory T-cell number, phenotype, or function may reduce local tolerance and permit unchecked inflammation. The balance between effector T-cell expansion and regulatory control may therefore be more informative than the presence of any single subset (10, 31, 35). This balance may also differ between peripheral blood, bronchoalveolar lavage fluid, and lung tissue, which is one reason why blood-based markers alone may incompletely represent pulmonary immune activity (44, 56, 67).

### 4.2 Myeloid cell dysfunction

Myeloid cells appear to shape both the intensity and persistence of CIP. Resident alveolar macrophages normally contribute to immune homeostasis, clearance of inhaled particles, and repair of epithelial injury (10, 31). In CIP, several studies suggest depletion or functional alteration of resident macrophage populations, accompanied by expansion of inflammatory monocytes or monocyte-derived macrophages. This shift may transform the alveolar space from a tolerogenic environment into one that supports cytokine amplification and antigen presentation (10, 29, 60).

Pro-inflammatory monocytes may produce mediators such as IL-1beta, TNF, IL-6, and IL-23, thereby linking innate immune activation to T-cell polarization (32, 56). This is particularly important in Th17.1- and GM-CSF-associated models of CIP, where monocytes and T cells may reinforce each other through reciprocal signaling (29, 65). Dendritic cells may also contribute by enhancing antigen presentation and sustaining T-cell activation (31, 60). These observations support a model in which CIP is not simply T-cell toxicity, but a multicellular inflammatory state involving both adaptive and innate immune compartments (**Figure 1**) (10, 29, 35).

**Figure 1:**
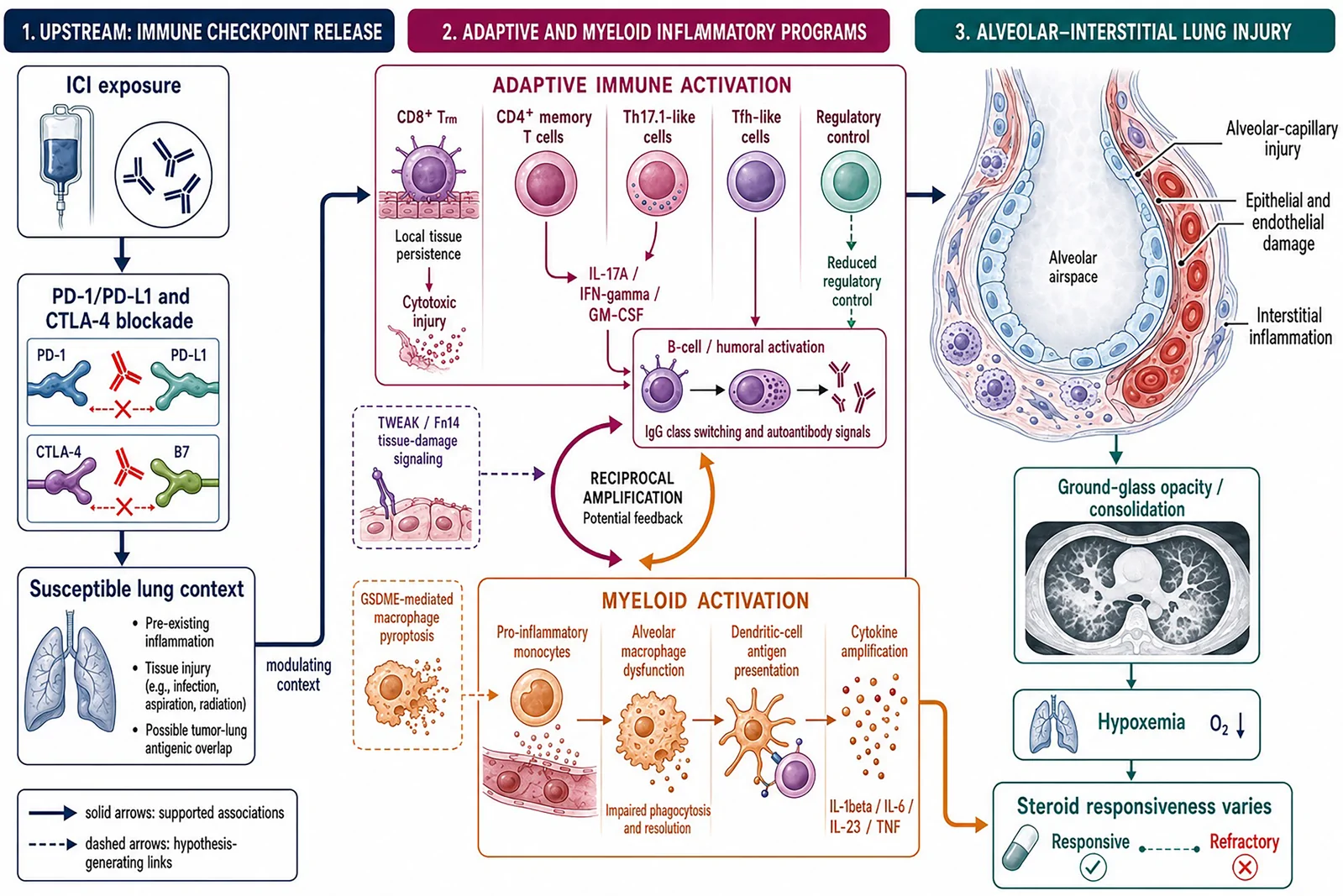
Multilevel immunopathogenesis of CIP. Immune checkpoint inhibitor (ICI) exposure, particularly blockade of the PD-1/PD-L1 and CTLA-4 pathways, may release inhibitory constraints on pulmonary immune responses in a susceptible lung context. The schematic summarizes the proposed interaction between adaptive immune activation, myeloid-cell dysfunction, cytokine amplification, and tissue injury. The adaptive immune compartment includes CD8+ tissue-resident memory T cells, CD4+ memory T cells, Th17.1-like cells, Tfh-like cells, and reduced regulatory control. These populations may contribute to local cytotoxic injury, persistent inflammation, antigen-driven immune activation, and humoral responses. The myeloid compartment includes pro-inflammatory monocytes, dysfunctional alveolar macrophages, enhanced dendritic-cell antigen presentation, and inflammatory mediators such as IL-1beta, IL-6, IL-23, TNF, IL-17A, IFN-gamma, and GM-CSF. Reciprocal signaling between activated T cells, myeloid cells, and B-cell/humoral pathways may amplify inflammation within the alveolar and interstitial compartments. These processes can converge on epithelial and endothelial damage, alveolar-capillary injury, radiographic abnormalities such as ground-glass opacity or consolidation, hypoxemia, and variable corticosteroid responsiveness. GSDME-mediated macrophage pyroptosis and TWEAK/Fn14 tissue-damage signaling are shown as exploratory pathways. Solid arrows indicate associations supported by current clinical or translational observations, whereas dashed arrows indicate hypothesis-generating links. The arrows represent conceptual relationships rather than a fixed linear sequence, and the schematic does not imply that all pathways occur in every patient with CIP or that any individual immune population or cytokine is sufficient to diagnose CIP or predict treatment response.

Myeloid-cell dysfunction may also help explain differences in disease severity (33, 44). A limited, well-contained inflammatory response may resolve with corticosteroids, whereas broader recruitment of inflammatory monocytes, macrophage activation, or inflammasome-like tissue injury may be associated with more extensive lung damage. Some emerging studies have also suggested roles for macrophage pyroptosis and tissue-damage signaling pathways (35, 68).These mechanisms remain exploratory, but they are relevant because they provide a possible bridge between immune activation and the radiographic or clinical appearance of diffuse alveolar injury (26, 46, 68).

### 4.3 Humoral immunity and autoantibody responses

Although CIP has traditionally been viewed as a T-cell-dominant toxicity, humoral immunity may contribute in selected patients. B-cell activation, clonal expansion, immunoglobulin class switching, and autoantibody signals have been reported in broader immune-related adverse event research and in emerging CIP-specific studies (69, 70). Anti-CD74 antibodies and other pre-existing or treatment-emergent antibodies have been proposed as potential contributors or biomarkers of immune toxicity (70–72). At present, however, the evidence is insufficient to define a uniform antibody-mediated subtype of CIP (22, 35).

The value of humoral findings may lie in their ability to identify a subset of patients with sustained immune activation rather than acute inflammatory injury alone (60, 70, 71). Tfh-like cell enrichment, B-cell activation, and autoantibody production could reflect an antigen-driven process that persists beyond the initial checkpoint blockade effect (58, 70). Clinically, this may be relevant for relapsing or chronic CIP, but the interpretation requires caution because autoantibodies may be present without direct pathogenicity (16, 35). Future work should clarify whether humoral signatures predict recurrence, steroid dependence, or risk during ICI rechallenge (22, 37).

### 4.4 Cytokine and tissue-damage pathways

Cytokine profiling provides another layer for understanding inflammatory phenotypes. IL-17A, IFN-gamma, GM-CSF, IL-1beta, IL-6, IL-23, TNF, and CXCL10 have all been implicated in different studies or mechanistic models. These mediators should not be considered interchangeable inflammatory markers. IFN-gamma and CXCL10 may reflect Th1-like chemokine activation; IL-17A and IL-23 may point toward Th17-associated inflammation; GM-CSF may connect activated T cells with myeloid-cell recruitment; and IL-1beta, IL-6, and TNF may reflect innate immune amplification (29, 64, 65). In parallel, tissue-damage pathways such as TWEAK/Fn14 signaling and GSDME-mediated macrophage pyroptosis may contribute to alveolar injury in selected contexts (35, 68).

For clinicians, the key issue is whether these cytokine patterns can eventually inform management.At present, cytokine data remain largely exploratory and are not ready to replace clinical judgment. Nonetheless, they help frame why second-line immunomodulatory strategies might not be equivalent across patients. A patient with predominantly Th17.1-myeloid activation may not be biologically identical to a patient with diffuse cytotoxic T-cell-mediated injury or chronic humoral activation (29, 30, 35). This distinction is clinically appealing, but prospective validation is needed before cytokine-guided treatment can be adopted as routine practice (22, 34, 54).

### 4.5 Linking immune phenotypes to clinical features

The central translational challenge is to connect immune phenotypes with clinical decisions (**Table 2**). CD8+ tissue-resident memory T-cell expansion may be associated with local tissue injury, persistence, or recurrence. Th17.1, GM-CSF, and IL-23 activation may suggest a more persistent inflammatory program and possible steroid resistance. Pro-inflammatory monocyte expansion may contribute to rapid progression and systemic inflammatory features. Macrophage activation or pyroptosis may amplify alveolar damage, while Tfh-like and B-cell activation may indicate sustained antigen-driven immune responses (30, 68, 70).

**Table 2:** Immune-inflammatory programs and their translational meaning in CIP.

Immune axis	Key CIP signal	Clinical reading	Evidence limit
CD4+ memory T-cell enrichment	Expanded CD4+ memory-like T cells in BALF; local immune activation.	May indicate a persistent recall-like compartment and slower resolution.	Observational BALF data; not proof of pathogenicity.
Th17.1/GM-CSF/IL-23 axis	Th17.1-like cells with IFNG, IL17A, CSF2; monocyte crosstalk.	May define a self-amplifying loop and steroid-refractory tendency.	Hypothesis-generating; not prospectively validated.
CD8+ effector memory and tissue-resident memory T cells	Cytotoxic CD8+ cells, tissue residency, clonal expansion, possible antigen overlap.	May underlie epithelial injury and reactivation after ICI rechallenge.	Context dependent; cross-reactivity remains unproven.
Tfh-like and B-cell/humoral activation	Tfh-like cells, B-cell activation, class switching, autoantibody signals.	May reflect sustained antigen-driven immunity in relapsing or chronic cases.	Nonspecific; antibodies may be markers rather than mediators.
Pro-inflammatory monocyte and macrophage programs	Inflammatory monocytes and macrophages with IL-1B, IL-6, IL-23, TNF.	May amplify alveolar-interstitial injury and accelerate progression.	Severe-case sampling bias; no validated routine biomarker.
Alveolar macrophage dysfunction and pyroptotic injury	Loss or dysfunction of resident alveolar macrophages; exploratory GSDME pyroptosis.	May impair resolution and link immune activation to diffuse injury.	Mechanistic evidence is early; translation is unproven.
Cytokine and chemokine amplification	IL-17A, IFNG, GM-CSF, IL-1B, IL-6, IL-23, TNF, CXCL10.	May help separate lymphocytic, myeloid, Th17-skewed, and mixed states.	Pleiotropic and nonspecific; cytokine-guided care is premature.
Tissue-damage and repair signaling	TWEAK/Fn14, epithelial-endothelial injury, residual interstitial/fibrotic change.	May be relevant to incomplete recovery and long-term impairment.	Exploratory only; antifibrotic strategies remain investigational.

BALF, bronchoalveolar lavage fluid; CD, cluster of differentiation; CIP, checkpoint inhibitor pneumonitis; GM-CSF, granulocyte-macrophage colony-stimulating factor; IFN-gamma, interferon gamma; IgG, immunoglobulin G; IL, interleukin; Th, T helper; Tfh, T follicular helper; TNF, tumor necrosis factor; Trm, tissue-resident memory T cell; TWEAK/Fn14, tumor necrosis factor-like weak inducer of apoptosis/fibroblast growth factor-inducible 14.

The table summarizes immune-inflammatory programs as translational interpretive axes rather than validated diagnostic categories. No single immune-cell population, cytokine, or tissue-damage pathway is sufficient to diagnose CIP or guide corticosteroid and second-line treatment decisions without clinical, radiographic, and microbiological context.

These links remain probabilistic rather than deterministic. No single immune-cell population or cytokine can currently diagnose CIP, predict steroid response, or determine rechallenge safety on its own. However, when interpreted alongside clinical severity, radiographic pattern, oxygen requirement, inflammatory markers, and early treatment response, immune phenotyping can support a more nuanced framework. The goal is not to divide patients into rigid mechanistic categories, but to recognize biologically meaningful patterns that may explain clinical behavior and guide future risk-adapted management (35).

## 5 Clinical and radiographic phenotyping

Clinical and radiographic phenotyping provides a practical bridge between the biological heterogeneity of CIP and the decisions that must be made at the bedside. In routine care, clinicians rarely encounter a textbook presentation. Instead, they face a patient whose symptoms, oxygenation, imaging pattern, treatment history, and pace of progression together suggest a pulmonary immune toxicity that may or may not behave like the next case (11, 33, 42). For this reason, the value of phenotyping lies not in naming a radiographic pattern alone, but in clarifying what that pattern implies for severity, trajectory, and management (13, 46, 54).

### 5.1 Clinical phenotypes

An asymptomatic radiographic phenotype is often discovered incidentally during surveillance imaging. These cases may show subtle ground-glass opacities, mild patchy consolidation, or limited interstitial abnormalities without a clear clinical syndrome. Although the patient may initially appear stable, asymptomatic CIP should not be dismissed as trivial, because radiographic progression may occur before symptoms become obvious (19, 20). The most important clinical question is whether the abnormality is static, improving, or evolving toward symptomatic disease.

A mild symptomatic phenotype usually includes cough, exertional dyspnea, chest tightness, or low-grade fever without a major oxygen requirement. This is often the category in which diagnostic uncertainty is greatest, because symptoms overlap with infection, cancer-related complaints, treatment fatigue, or radiation effects. Mild symptoms do not necessarily imply mild biology, but mildly symptomatic/CTCAE grade 2 CIP generally warrants temporary ICI interruption, exclusion of alternative diagnoses, and consideration of oral corticosteroids when immune-mediated pneumonitis is favored, with close reassessment of symptoms, oxygenation, and imaging (21).

The hypoxemic inflammatory phenotype is more concerning. Here, dyspnea is accompanied by objective oxygen desaturation, increasing work of breathing, and more extensive parenchymal involvement. Patients may initially present outside the ICU but deteriorate quickly if the inflammatory process continues unchecked. This phenotype is clinically important because it is the point at which early treatment escalation, multidisciplinary input, and repeated reassessment become especially relevant (20, 54). In practice, the presence of hypoxemia often weighs more heavily than the formal toxicity grade alone (19, 22).

At the severe end of the spectrum is the diffuse alveolar injury phenotype, which can resemble acute lung injury or acute respiratory distress syndrome. These cases are characterized by extensive bilateral opacities, marked hypoxemia, and a rapidly progressive course that may require high-flow oxygen, ventilatory support, or intensive care. The biological significance of this phenotype is different from that of limited organizing pneumonia-like disease. A diffuse injury pattern suggests that the process is no longer confined to a small inflammatory niche and may reflect broad alveolar involvement, macrophage activation, epithelial damage, or steroid-refractory inflammation (17, 29).

Finally, a chronic or relapsing phenotype deserves explicit attention. Some patients improve initially but develop recurrent symptoms or persistent radiographic abnormalities during corticosteroid tapering. Others transition into a state of residual interstitial change, fibrotic remodeling, or prolonged treatment dependence. These cases are easy to underestimate if the clinician focuses only on the first acute episode (16, 23). A chronic course should prompt consideration of whether the inflammatory process has not fully resolved, whether an alternative diagnosis has been missed, or whether a distinct steroid-dependent phenotype is present (16, 35).

### 5.2 Radiographic patterns

Radiographic pattern recognition remains central to the diagnostic work-up, but no single pattern is pathognomonic for CIP. Organizing pneumonia is one of the more common appearances and is often characterized by peripheral or peribronchovascular patchy consolidation and ground-glass opacity, and it may also be associated with migratory or waxing-and-waning radiographic changes. In the right clinical setting, this pattern may correspond to a more steroid-responsive phenotype, although exceptions are common. Nonspecific interstitial pneumonia-like disease may present with diffuse or bilateral ground-glass change, reticulation, and lower-lobe predominance, sometimes suggesting a broader interstitial process (13, 45).

Hypersensitivity pneumonitis-like patterns usually appear as multifocal ground-glass opacities, centrilobular nodularity, and patchy distribution, sometimes with relative sparing of the lung bases. This appearance may raise concern for immune-mediated inflammation but also overlaps with infection or other drug-related lung injury. At the severe end, diffuse alveolar damage or ARDS-like patterns are associated with extensive bilateral opacities, rapid clinical deterioration, and a more guarded prognosis (17, 26, 46). In such cases, the radiographic pattern is not simply descriptive; it is often a warning sign that the inflammatory burden is high and the margin for delay is small (22, 46).

Ground-glass opacity and consolidation are the most common building blocks across these patterns, but the relative distribution, symmetry, and extent are often more informative than density alone. Peripheral, focal, or limited multifocal disease may be easier to follow longitudinally and may permit careful outpatient management in selected patients. In contrast, diffuse or rapidly spreading bilateral involvement should prompt greater concern for severe CIP, infection, or another overlapping pulmonary process. Although CTCAE incorporates both symptoms and radiographic extent, distribution alone should be interpreted as a modifier of clinical concern rather than a standalone severity determinant, because its prognostic meaning is most meaningful when it translates into symptoms, oxygen requirement, or physiologic decline (17, 28, 50). Residual fibrotic changes after apparent clinical recovery can also be clinically meaningful, because they may indicate incomplete resolution and impaired pulmonary reserve, particularly when they are accompanied by persistent dyspnea or declining pulmonary function; whether they independently predispose to recurrent pneumonitis after ICI rechallenge remains uncertain (16, 36).

### 5.3 Radiographic disease burden

Beyond pattern recognition, disease burden matters. The amount of lung involved, the speed of radiographic progression, and whether abnormalities are unilateral or bilateral all influence clinical risk. A small lesion in a symptomatic patient may carry a different implication from diffuse opacification in a patient with only mild dyspnea, and both the absolute extent and the trajectory should be considered. Serial imaging is therefore more informative than a single scan whenever the clinical situation permits (19, 23).

Radiographic burden should also be interpreted in the context of baseline lung status (14, 42, 43). A patient with prior interstitial abnormalities, emphysema, or post-radiation changes may have less reserve than the scan alone suggests. In such settings, even modest new abnormalities can produce disproportionate physiological effects. Conversely, a patient with extensive-appearing changes on imaging may remain clinically stable if the abnormalities are slowly evolving and the underlying lung reserve is preserved (13, 23). The relationship between imaging severity and physiological severity is therefore important but not linear (22, 46).

### 5.4 Differential diagnosis

The radiographic phenotype of CIP is useful only if alternative diagnoses are actively considered. Infection remains the most important competing diagnosis, particularly in patients receiving corticosteroids, chemotherapy, or combined immunotherapy (28, 55). Viral, bacterial, and fungal causes may mimic or coexist with CIP, and a partial early response to steroids does not reliably exclude infection (55). Radiation pneumonitis is another major consideration because it may share both symptoms and imaging features, especially in previously irradiated lung regions (50).

Tumor progression, lymphangitic spread, pulmonary embolism, drug-induced pneumonitis, acute exacerbation of pre-existing interstitial lung disease, and diffuse alveolar hemorrhage also belong in the differential diagnosis. The practical implication is that the radiographic appearance should not be treated as a diagnosis in itself. Rather, it should be read as one piece of a broader clinical argument that includes timing, symptoms, oxygen requirement, prior therapies, microbiology, and response to initial treatment (11, 12, 19). This interpretive approach is especially important in lung cancer, where multiple pulmonary insults often overlap (11, 12, 41).

Taken together, clinical and radiographic phenotyping does not replace immune phenotyping; it complements it. Symptoms, oxygenation, distribution, extent, and progression rate provide the visible clinical layer of CIP heterogeneity, while later sections of this review address the immune mechanisms that may underlie those differences (15, 29, 30). The goal is to use phenotype recognition to move from a generic label toward a more precise and clinically useful understanding of the patient in front of us (23, 54). This clinical-radiographic phenotype discussion sets the stage for the four-dimensional stratification framework presented in Section 6.

## 6 A four-dimensional stratification framework for CIP

The main clinical problem in CIP is not simply whether pneumonitis is present, but what kind of pneumonitis is present and how that phenotype is likely to behave. A binary diagnosis of CIP is useful for communication, but it does not capture the full range of severity, biological activity, or treatment responsiveness. For this reason, we propose a four-dimensional stratification framework that integrates clinical severity, radiographic phenotype, inflammatory and immune phenotype, and corticosteroid responsiveness (**Figure 2**) (18, 22, 54). The purpose of this framework is pragmatic: to move from a generic label toward a bedside-oriented understanding of risk, trajectory, and therapeutic urgency (19, 23).

**Figure 2:**
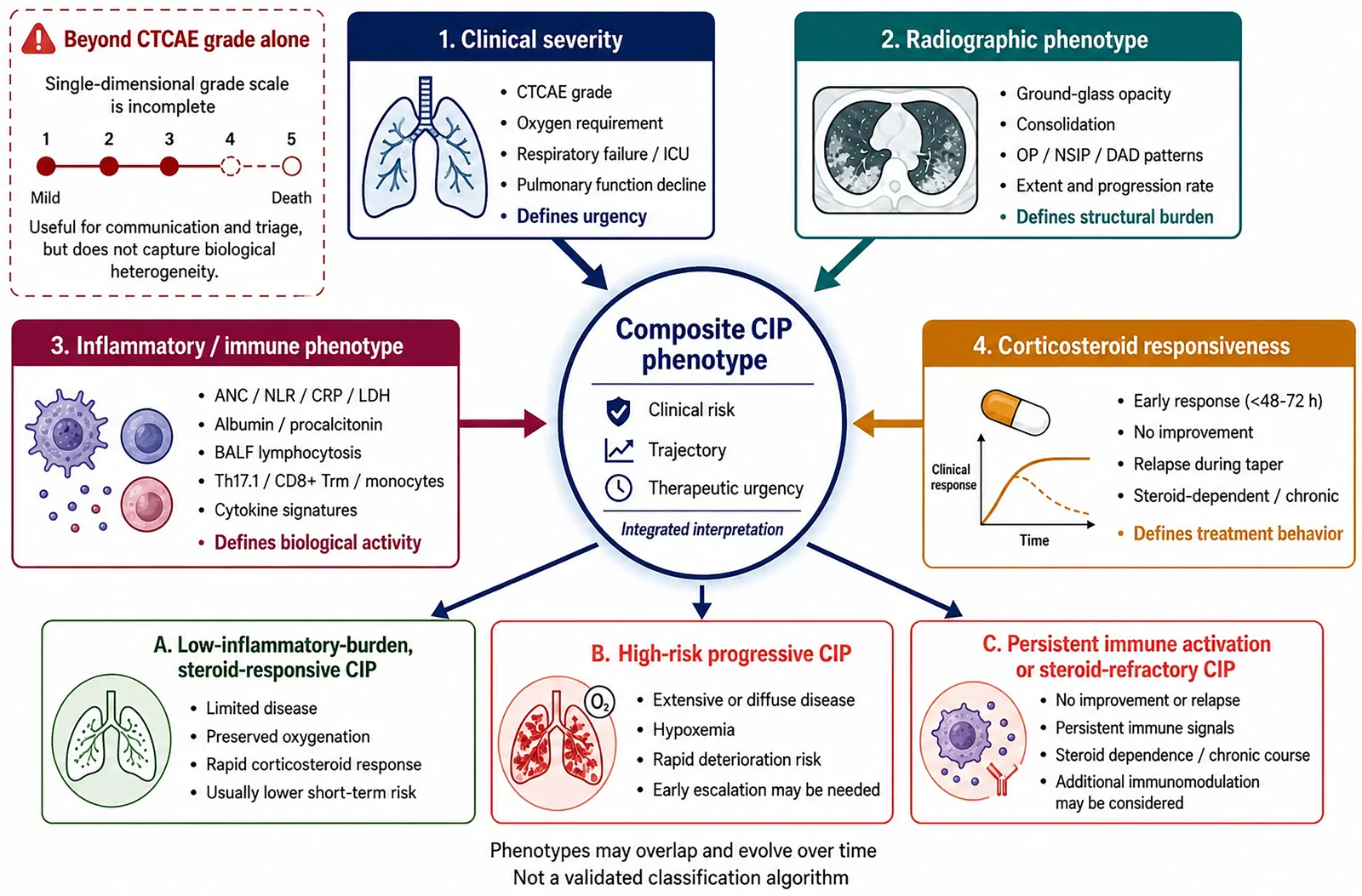
Four-dimensional phenotype framework for CIP. This schematic shows why CTCAE grade alone is insufficient to capture the biological heterogeneity of checkpoint inhibitor pneumonitis (CIP). Although CTCAE grade remains useful for communication and triage, the review proposes a composite framework that integrates four complementary dimensions: clinical severity, radiographic phenotype, inflammatory and immune phenotype, and corticosteroid responsiveness. Clinical severity incorporates CTCAE grade, oxygen requirement, respiratory failure or ICU need, and pulmonary function decline, and defines urgency. The lung icon in the clinical severity panel is a schematic representation of the respiratory system and is not intended to depict a specific anatomical lesion or disease pattern. Radiographic phenotype captures ground-glass opacity, consolidation, organizing pneumonia, nonspecific interstitial pneumonia, diffuse alveolar damage, extent, and progression rate, and defines structural burden. Inflammatory and immune phenotype integrates accessible markers such as ANC, NLR, CRP, LDH, albumin, procalcitonin, BALF lymphocytosis, and immune-cell signatures including Th17.1, CD8+ tissue-resident memory T cells, and pro-inflammatory monocytes, and defines biological activity. Corticosteroid responsiveness captures early improvement, non-response, relapse during tapering, and steroid dependence or chronicity, and defines treatment behavior. These four dimensions converge on a composite CIP phenotype that informs clinical risk, trajectory, and therapeutic urgency. The framework yields three broad clinical phenotypes: low-inflammatory-burden, steroid-responsive CIP; high-risk progressive CIP; and persistent immune-activation or steroid-refractory CIP. Phenotypes may overlap and evolve over time, and the framework should be viewed as a clinically grounded heuristic rather than a validated classification algorithm.

The evidence supporting the four dimensions is heterogeneous and should not be interpreted as equally validated across domains. Clinical severity and radiographic phenotype are supported primarily by established clinical assessments, imaging observations, guidelines, and cohort studies. Inflammatory and immune phenotyping is informed by accessible biomarkers, BALF findings, tissue-based studies, cytokine analyses, and emerging immune-profiling approaches. Corticosteroid responsiveness is based mainly on longitudinal clinical behavior and treatment-response observations and remains less standardized. Accordingly, the proposed framework should be viewed as a clinically grounded conceptual synthesis and hypothesis-generating model rather than a validated score or predictive classification algorithm.

Although the four dimensions are presented within one integrated framework, they are not necessarily assessed at the same time. Initial phenotype or risk assessment is based primarily on clinical severity, radiographic phenotype, and inflammatory and immune features that can be evaluated at presentation. Corticosteroid responsiveness is a longitudinal treatment-response dimension that can only be assessed after corticosteroid therapy has been initiated and during subsequent follow-up. It should therefore be interpreted as a dynamic modifier of the initial phenotype rather than as a simultaneous baseline characteristic. The framework integrates these domains across the clinical course, linking initial risk stratification with treatment response and later clinical evolution.

### 6.1 Dimension 1: clinical severity

Clinical severity remains the first and most immediate layer of assessment. CTCAE grade provides a useful starting point, but it should be interpreted together with oxygen requirement, respiratory rate, oxygen saturation, need for hospitalization, and whether the patient requires high-flow oxygen or mechanical ventilation. Pulmonary function decline and ICU admission are also important markers of severity, particularly in patients whose baseline pulmonary reserve is limited (14, 42, 43). In practice, a severity grade should be treated as a snapshot rather than a complete description of disease biology (17, 22, 33).

The value of this dimension is that it defines urgency. A patient with minor symptoms and preserved oxygenation can often undergo closer outpatient follow-up, whereas a patient with progressive hypoxemia or rising respiratory support needs requires rapid escalation and repeated reassessment. However, severity alone cannot distinguish a mild but biologically active inflammatory process from a severe presentation that is already entering a steroid-refractory or diffuse injury phase (17, 26, 29).

### 6.2 Dimension 2: radiographic phenotype

Radiographic phenotype adds structural information that severity grading cannot provide. Disease distribution, ground-glass opacity, consolidation, organizing pneumonia, NSIP-like change, diffuse alveolar damage, and the pace of radiographic progression are all clinically meaningful. They help the clinician estimate whether the process is focal or diffuse, peripheral or central, slowly evolving or rapidly spreading (13, 46). These patterns are not absolute entities, but they do represent recurring imaging grammars that often correspond to different clinical trajectories (15, 46).

For example, limited organizing pneumonia-like disease often has a more favorable short-term course than diffuse alveolar damage-like disease, although exceptions are common. A bilateral, diffuse, or rapidly progressive pattern usually raises concern for more extensive alveolar involvement and a narrower therapeutic window (17, 26, 46). Radiographic phenotype is therefore not just descriptive; it is an early clue to the likely biological burden of the lung injury and to the intensity of monitoring that will be required (22, 45, 46).

### 6.3 Dimension 3: inflammatory and immune phenotype

The third dimension addresses the underlying immune activity that may drive clinical behavior. Accessible variables include absolute neutrophil count, neutrophil-to-lymphocyte ratio, CRP, LDH, procalcitonin, and albumin. More specialized data include IL-17A, IL-6, IL-1beta, and other cytokines, as well as bronchoalveolar lavage lymphocytosis and immune-cell signatures involving Th17.1, Tfh-like, CD8+ tissue-resident memory, and pro-inflammatory monocyte populations (29, 56, 73). These markers are not interchangeable, and no single one can currently define CIP. Their value lies in pattern recognition (30, 31).

An inflammatory phenotype characterized by high neutrophil burden, low albumin, elevated cytokines, and strong myeloid-cell activation may point toward more aggressive disease, whereas a phenotype dominated by local lymphocytic activation with limited systemic inflammation may behave differently. Th17.1- or GM-CSF-skewed inflammation may be particularly relevant when early corticosteroid response is poor (17, 29, 65). In contrast, a phenotype that shows relatively modest inflammatory burden and limited immune activation may be more likely to resolve with standard therapy.At present, these distinctions are best viewed as biologically plausible and clinically useful hypotheses rather than validated classification rules (22, 35).

### 6.4 Dimension 4: corticosteroid responsiveness

Corticosteroid responsiveness is the most clinically actionable dimension and may be the one that most directly links phenotype to management. Some patients improve quickly after corticosteroid initiation, with reduced symptoms and stable or improving oxygenation within days. Others fail to improve within 48 to 72 hours, relapse during tapering, become steroid dependent, or evolve into a chronic fibrotic state (16–18). These patterns are not merely treatment outcomes; they are phenotype-defining features (33).

Early response often supports a lower-inflammatory-burden phenotype, although it does not exclude future relapse.Lack of response should trigger a more deliberate search for infection, alternative diagnoses, inadequate steroid intensity, or a more refractory inflammatory program. Relapse during tapering may indicate persistent immune activation rather than incomplete initial treatment alone (16). A chronic fibrotic phenotype is particularly important because it can leave the patient with long-term respiratory limitation even after the acute phase has passed (36).

Corticosteroid responsiveness should be assessed longitudinally rather than at the initial presentation. Steroid-refractory CIP refers to failure to show meaningful clinical or oxygenation improvement after adequate initial corticosteroid therapy, after infection, alternative diagnoses, treatment intensity, and other potentially reversible factors have been reconsidered. Steroid-relapsing CIP refers to recurrence after an initial response during corticosteroid tapering. Steroid-dependent CIP refers to disease that cannot be controlled during corticosteroid withdrawal or requires ongoing corticosteroid therapy to maintain clinical stability. These patterns may overlap but represent different treatment trajectories. Chronic or fibrotic CIP should be considered separately because persistent interstitial abnormalities or functional impairment do not necessarily indicate ongoing steroid-responsive inflammation.

### 6.5 Proposed clinical phenotypes

Taken together, these four dimensions support three broad clinical phenotypes. The first is low-inflammatory-burden, steroid-responsive CIP. These patients usually have low-grade symptoms or limited oxygen requirement, restricted radiographic involvement, modest inflammatory activity, and rapid improvement after corticosteroids.They often represent the group in which short-term prognosis is best, although they still require follow-up to ensure radiographic resolution and safe tapering (19, 23).

The second phenotype is high-risk progressive CIP. These patients typically have grade 3 to 4 disease, extensive or diffuse radiographic involvement, significant hypoxemia, and a higher likelihood of rapid deterioration. Baseline interstitial lung disease, prior thoracic radiotherapy, or markedly abnormal inflammatory markers may further increase concern (42). The key issue in this phenotype is not only treatment intensity, but timing: delay can be more consequential here than in lower-risk cases (21, 54).

The third phenotype is persistent immune-activation or steroid-refractory CIP. These patients either do not improve after adequate corticosteroid therapy or relapse during tapering, suggesting that the inflammatory process remains active despite initial treatment. They may exhibit Th17.1, CD8+ Trm, pro-inflammatory monocyte, or sustained cytokine signatures, although these are not required for the phenotype to be clinically recognized (29, 30, 56). This is the group in which escalation beyond corticosteroids is most likely to be considered, but also the group in which uncertainty regarding infection, alternative lung injury, and the safety of further immunosuppression is greatest (28, 55).

### 6.6 How the framework should be used

The framework is meant to be integrated, not applied mechanistically. A patient can have severe clinical disease but limited radiographic spread, or extensive imaging abnormalities with preserved oxygenation, or strong inflammatory signals with only modest symptoms. No single dimension should override the others (22, 54). Instead, the goal is to create a composite impression of risk and behavior that is more informative than CTCAE grade alone (24, 34).

In practical terms, the four dimensions can be viewed as answering four bedside questions. How sick is the patient now? What does the lung look like? How inflamed is the process likely to be? Is the disease responding to corticosteroids as expected? When these questions are answered together, the clinician is better positioned to decide whether observation, corticosteroids, escalation of immunomodulation, or reconsideration of the diagnosis is most appropriate (**Figure 3**) (20, 21).

**Figure 3:**
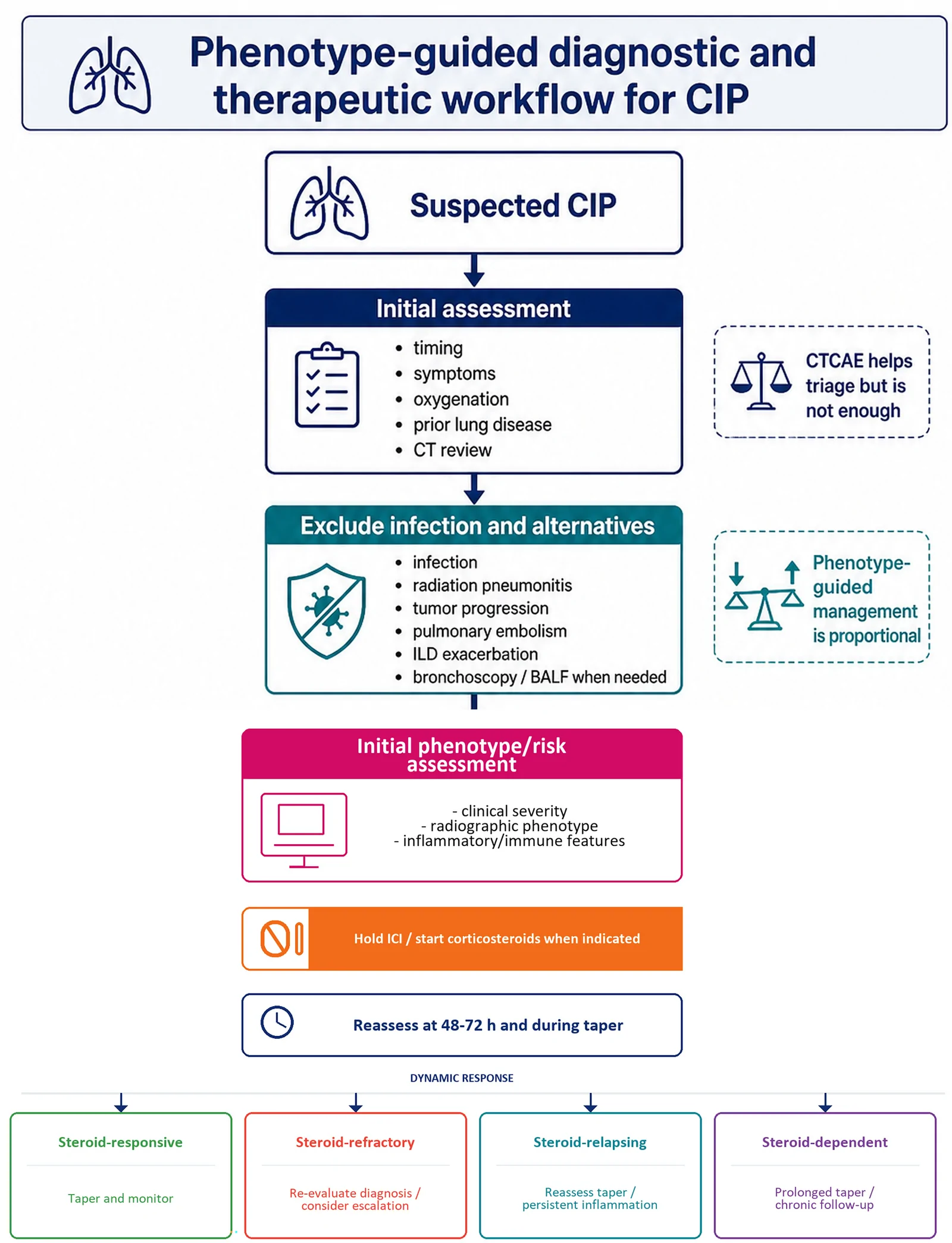
Phenotype-guided diagnostic and therapeutic workflow for CIP. schematic shows a temporally ordered bedside decision pathway for suspected CIP. Initial assessment incorporates timing, symptoms, oxygenation, prior lung disease, and CT review. Alternative causes are evaluated, including infection, radiation pneumonitis, tumor progression, pulmonary embolism, and ILD exacerbation, with bronchoscopy/BALF when clinically indicated. Initial phenotype/risk assessment then integrates clinical severity, radiographic pattern and burden, and available inflammatory/immune findings. Based on this initial assessment, ICI therapy is held and corticosteroids are initiated when clinically appropriate. Corticosteroid responsiveness is assessed only after treatment initiation, including reassessment at 48–72 hours and monitoring during tapering. Early improvement supports a steroid-responsive trajectory; lack of meaningful improvement after adequate therapy prompts diagnostic reassessment and consideration of steroid-refractory disease; recurrence during tapering suggests steroid-relapsing disease; and inability to discontinue corticosteroids without recurrence suggests steroid-dependent disease. Improvement leads to tapering and monitoring, whereas persistent or recurrent disease prompts diagnostic re-evaluation, reassessment of the taper, consideration of escalation, or chronic follow-up according to the response pattern. CTCAE helps with initial triage but is not sufficient on its own. The workflow is intended to represent a temporally ordered, phenotype-guided approach rather than a rigid treatment algorithm.

This framework also provides a conceptual bridge to the next sections of the review. The diagnostic approach should be phenotype-aware, because the probability of infection, progression, or steroid-refractory disease changes across strata (12, 28, 55). Likewise, therapy should be phenotype-guided, because not every patient with CIP requires the same intensity, duration, or type of immunosuppression. In this sense, the four-dimensional framework is not a theoretical exercise. It is the organizing logic for the rest of the review (18, 22, 35).

## 7 Phenotype-guided diagnostic framework

The diagnostic workflow for suspected CIP is summarized in **Figure 3**. The clinician should integrate timing, symptoms, oxygenation, high-resolution CT pattern and burden, exclusion of infection and alternative diagnoses, selective bronchoscopy/BALF when needed, and multidisciplinary review to determine whether the working diagnosis should remain broad or move toward phenotype-guided management (20, 21, 54).

### 7.1 Initial clinical assessment

The first step is careful clinical reconstruction. Timing of ICI exposure remains important, although no single time window excludes CIP. New respiratory symptoms after treatment initiation, especially when they emerge without another obvious explanation, should prompt consideration of immune-related lung injury. Symptoms and oxygenation provide the immediate severity signal, while prior lung disease, thoracic radiotherapy, recent infectious exposure, and tumor status help define the background against which the lung injury is occurring. Baseline pulmonary function is also important because the same absolute degree of lung injury may be tolerated very differently depending on pre-existing reserve (14, 42, 43).

This initial assessment should be framed as a probability problem rather than a definitive diagnosis. A patient with compatible timing, worsening dyspnea, and new bilateral infiltrates has a higher pre-test probability of CIP than a patient with stable symptoms and a focal abnormality in a previously irradiated field (13). The purpose of the initial assessment is therefore to determine whether the working diagnosis should remain broad or shift toward more aggressive evaluation and treatment (19, 21, 54).

### 7.2 Radiographic assessment

High-resolution chest CT is the central imaging study in suspected CIP. It provides the most useful information on distribution, density, extent, and progression of lung involvement. Serial imaging is often more informative than a single scan because the pace of change can reveal whether the process is stable, inflammatory, or rapidly progressive (19, 23). Pattern recognition should focus on organizing pneumonia-like, nonspecific interstitial pneumonia-like, hypersensitivity pneumonitis-like, or diffuse alveolar damage-like appearances, but the clinician should not expect a perfectly pure pattern in every case (13, 45, 46).

Radiographic assessment should also address burden rather than pattern alone. A small volume of disease in a symptomatic patient may be clinically significant, while diffuse bilateral opacities in a patient with only mild dyspnea may still signal an unstable process. Rate of progression is particularly important because rapidly expanding abnormalities should lower the threshold for hospitalization, bronchoscopy, and treatment escalation (17, 33). Imaging should therefore be interpreted as a dynamic marker of disease behavior, not as a static label (13, 23).

### 7.3 Exclusion of infection and alternative diagnoses

Because CIP is a diagnosis of exclusion in many real-world situations, active evaluation for alternative causes is essential. Microbiological testing should be tailored to the clinical scenario and may include bacterial, viral, and fungal investigations, especially in patients receiving corticosteroids or cytotoxic therapy. A partial response to steroids does not reliably exclude infection, and infection can coexist with immune-mediated lung injury (28, 55).For that reason, diagnostic confidence should increase only when the clinical trajectory, imaging, and laboratory data are internally consistent (22, 35).

Radiation pneumonitis is another major competing diagnosis, particularly when abnormalities are geographically aligned with prior radiation fields. Tumor progression, lymphangitic carcinomatosis, pulmonary embolism, drug-induced pneumonitis, acute exacerbation of underlying interstitial lung disease, and diffuse alveolar hemorrhage should also remain on the differential list (12, 19, 23). The key diagnostic task is not to exclude every alternative with equal intensity, but to identify the diagnoses that would materially change management if missed (19, 21, 54). In a lung cancer population, this threshold is low because several overlapping pulmonary processes may be present at the same time (11, 28, 41).

### 7.4 Role of BALF and bronchoscopy

Bronchoscopy with bronchoalveolar lavage can be valuable when the diagnosis remains uncertain or when infection cannot be safely excluded on clinical grounds alone. Its role is primarily to support exclusion of infection, obtain differential cell counts, and identify a lymphocytic or mixed inflammatory pattern that may be compatible with CIP (41, 60, 67). BALF can also provide material for research-oriented immune profiling, although such analyses are not yet standardized for routine clinical use (29, 30, 56). These BALF findings should be interpreted alongside the inflammatory and immune phenotyping discussed earlier in the manuscript. In parallel, routinely available serum markers such as ANC, NLR, CRP, LDH, albumin, and procalcitonin can support bedside assessment, whereas cytokine profiling and other research-oriented immune assays remain less widely available and are not yet standardized for routine clinical use.

BALF findings should be interpreted carefully. Lymphocytosis may support an inflammatory pneumonitis phenotype, but it is not specific for CIP and does not exclude other causes. Likewise, the absence of striking BALF abnormalities does not rule out immune-mediated disease, particularly if corticosteroids have already been started or if sampling is delayed. The main clinical value of BALF is therefore complementary rather than definitive: it can strengthen the diagnostic argument, reduce uncertainty, and guide the level of confidence with which immunosuppression is continued or escalated (19, 55, 56).

### 7.5 Multidisciplinary decision-making

Phenotype-guided diagnosis is inherently multidisciplinary. Medical oncology, pulmonology, radiology, infectious diseases, and critical care medicine each contribute a different part of the diagnostic picture, and radiation oncology may be needed when prior thoracic radiotherapy complicates interpretation (12, 50). The value of multidisciplinary review is not simply consensus for its own sake; it is the integration of timing, imaging, microbiology, and treatment response into a single decision about whether the patient has likely CIP and how urgently intervention is needed (19, 23).

This approach also reduces the risk of diagnostic anchoring. If the team assumes that all pulmonary infiltrates after ICI exposure are CIP, infection or tumor progression may be missed. If the team assumes the opposite, effective treatment may be delayed. A phenotype-guided framework helps balance these risks by making the diagnostic reasoning explicit. The result is a working diagnosis that is proportional to the available evidence and flexible enough to be revised as new information emerges (23, 54).

In sum, the diagnostic framework proposed here is not intended to create an artificial algorithm for every patient. Its purpose is to organize bedside reasoning around phenotype, urgency, and competing diagnoses. That logic sets the stage for treatment decisions in the next section, where initial corticosteroid therapy, escalation strategies, and management of refractory or chronic disease are considered in relation to the phenotype that has been established (17, 18).

## 8 Phenotype-guided therapeutic strategies

Therapy for CIP should be guided by phenotype, severity, and early response rather than by a single rigid algorithm. The practical decision nodes are integrated into the text and **Figure 3**.The same diagnosis can justify very different interventions depending on whether the patient has mild radiographic disease, progressive hypoxemia, diffuse alveolar injury, or persistent inflammation after initial treatment. In practice, treatment decisions must balance three competing priorities: controlling pulmonary inflammation, avoiding unnecessary immunosuppression, and preserving the oncologic benefit of ICI therapy when possible. The greatest incremental value of the four-dimensional framework emerges in grade 3–4 disease and steroid-refractory CIP, where CTCAE alone is least sufficient and where earlier clinical, radiographic, and biomarker features help guide escalation (10, 19, 23).

Although CTCAE grade remains an important starting point for triage and communication, treatment decisions should be refined by the other dimensions of the framework. Observation after temporary ICI interruption may be reasonable in selected patients with limited and stable radiographic abnormalities, preserved oxygenation, low apparent inflammatory burden, and no evidence of progression. In contrast, systemic corticosteroid treatment should be considered more strongly when radiographic involvement is extensive or progressive, oxygenation worsens, inflammatory or immune activity appears substantial, or the clinical trajectory is unstable, even when the initial CTCAE grade is relatively modest. The intensity of treatment and need for hospitalization should be determined by the combined assessment of oxygen requirement, respiratory support needs, radiographic extent and progression, pulmonary reserve, and inflammatory activity. Persistent symptoms or oxygen requirements after initial treatment, relapse during corticosteroid tapering, or evidence of sustained immune activation should prompt diagnostic reassessment and consideration of escalation beyond corticosteroids, after infection and other alternative causes have been reconsidered. Thus, the four-dimensional framework is intended to refine grade-based management by linking current severity with structural burden, biological activity, and treatment behavior rather than to replace CTCAE grading with a separate rigid algorithm.

### 8.1 Low-risk and grade 1 disease

Patients with asymptomatic grade 1 disease often have limited radiographic involvement and preserved oxygenation. In this setting, close clinical and radiographic monitoring may be sufficient, particularly if the abnormalities are small, stable, and not clearly progressive. Temporary interruption of ICI is often reasonable while the diagnosis is clarified and follow-up imaging is arranged (19, 21). Corticosteroids are not automatically required for every low-grade case.The key is to avoid overtreatment while maintaining a low threshold to escalate if the trajectory changes (22, 54).

Even apparently mild disease should not be treated casually. Serial reassessment of symptoms, oxygenation, and imaging is important because some low-grade presentations evolve rapidly (19, 23). If the clinical picture becomes less stable, management should be reclassified according to the new phenotype rather than the initial grade alone (18, 33).

### 8.2 Grade 2 disease

Grade 2 CIP usually requires a more active approach. ICI therapy should be held, infection should be actively excluded, and systemic corticosteroids are generally appropriate when the clinical and radiographic picture supports immune-mediated pneumonitis.The response to treatment should be reassessed within 48 to 72 hours, because early improvement provides important information about both diagnosis and phenotype (17, 18, 54). A clear clinical response supports a steroid-responsive inflammatory process, whereas persistent symptoms or oxygen needs should prompt reconsideration of the working diagnosis or escalation of care (17, 28, 55).

Steroid tapering deserves special attention in grade 2 disease because some patients relapse during dose reduction even after initial improvement. This pattern suggests that the inflammatory process has not fully resolved and that taper speed may need to be adjusted (16, 18). From a practical standpoint, grade 2 CIP is often the point at which the clinician learns whether the disease behaves like a self-limited inflammatory event or a more persistent immune syndrome (17, 18, 35).

### 8.3 Grade 3–4 disease

Grade 3 to 4 CIP is a medical urgency. These patients usually require hospitalization, oxygen support, high-dose intravenous corticosteroids, and close monitoring for respiratory failure. In many cases, ICI should be permanently discontinued or suspended, depending on oncologic context and the certainty of the diagnosis. Early involvement of pulmonology, critical care, radiology, and infectious diseases is often appropriate because the differential diagnosis is broad and the consequences of delay can be severe (12, 19, 54).

In this phenotype, the therapeutic goal is not only to reduce inflammation but to prevent further alveolar damage (17, 26, 46). Secondary infection must be actively considered, particularly once corticosteroids have been started. A patient who fails to improve promptly should not simply be observed indefinitely; rather, the team should re-evaluate the phenotype, the competing diagnoses, and the need for second-line immunomodulation (17, 19, 35). High-grade disease is the setting in which phenotype-guided escalation is most relevant, but also the setting in which treatment risks are highest (17, 33, 54).

### 8.4 Steroid-refractory CIP

Steroid-refractory CIP is the most difficult therapeutic phenotype. Available evidence for second-line agents such as intravenous immunoglobulin, mycophenolate mofetil, TNF inhibition, IL-6 pathway inhibition, IL-17/IL-23 pathway inhibition, JAK inhibition, and other investigational immunomodulators is largely retrospective and based on small case series or selected clinical experience (**Table 3**). These agents should therefore be viewed as options for carefully selected patients rather than as established standard therapy (17, 54, 68).

**Table 3:** Evidence for immunomodulatory therapies in steroid-refractory or relapsing CIP.

Therapy class	Why it is considered	Evidence in CIP	Key caution
Intravenous immunoglobulin	Broad rescue immunomodulation when inflammation persists or infection remains difficult to exclude.	Reported in small series and case-based rescue strategies, usually with corticosteroids.	Response is inconsistent; evidence remains sparse.
Mycophenolate mofetil	Steroid-sparing suppression of lymphocyte activation and expansion.	Used in selected refractory or relapsing cases after inadequate steroid response.	Delayed onset and infection or cytopenia risk.
TNF inhibition	Targets cytokine amplification and macrophage-T-cell crosstalk.	Infliximab is the most frequently described rescue agent in published CIP experience.	Infection must be carefully excluded; outcomes are mixed.
IL-6 pathway inhibition	May fit hyperinflammatory phenotypes with systemic cytokine amplification.	Tocilizumab has been reported in isolated refractory CIP cases and small clinical experiences.	Evidence is limited; inflammatory markers may become harder to interpret.
IL-17/IL-23 pathway inhibition	Biologically aligned with Th17-skewed inflammation and GM-CSF-associated programs.	Supported mainly by mechanistic rationale and rare phenotype-informed use.	No prospective validation in CIP.
JAK inhibition	Broad downstream blockade of multiple cytokine signals.	Emerging rescue strategy in selected reports of refractory disease.	Infection risk and drug interactions remain important.
Combination or sequential rescue	Considered when a single agent is insufficient or phenotype remains unclear.	Used in highly selected cases, usually after multidisciplinary review.	Evidence is anecdotal and confounded by disease severity.

CIP, checkpoint inhibitor pneumonitis; GM-CSF, granulocyte-macrophage colony-stimulating factor; JAK, Janus kinase; TNF, tumor necrosis factor.

This table summarizes rescue immunomodulation for steroid-refractory or relapsing CIP. It is not a treatment algorithm, and all second-line decisions should be made only after reassessing infection, alternative diagnoses, and the evolving clinical phenotype.

The practical challenge is that steroid-refractory disease is defined clinically, but the underlying biology may differ from one patient to another. Some patients may have persistent T-cell-dominant inflammation, others may show stronger myeloid or cytokine activation, and still others may have occult infection or another overlapping lung injury. For that reason, second-line treatment should ideally be phenotype-informed, but it must remain evidence-limited (22, 35, 68). The choice of agent depends on the clinical scenario, trajectory, infection risk, prior treatment exposure, and institutional experience (17, 54). The clinician should also remain aware that intensifying immunosuppression may complicate infection control and may carry uncertain consequences for antitumor efficacy (7, 10, 19).

### 8.5 Chronic, relapsing, and fibrotic CIP

Chronic or relapsing CIP requires a different tempo of management. Some patients improve acutely but need prolonged corticosteroid tapering because symptoms or imaging abnormalities recur as the dose is reduced. Others develop persistent interstitial changes or residual functional impairment despite resolution of the initial inflammatory episode (16, 23).In these cases, the clinical task is no longer simply to suppress acute inflammation; it is to manage a longer-lasting pulmonary injury state (16, 23).

Prolonged tapering, pulmonary function monitoring, surveillance imaging, and attention to opportunistic infection are often appropriate in this setting (16, 19, 54). Residual fibrotic change may not respond in the same way as acute inflammation, and the clinician should avoid assuming that every persistent opacity reflects ongoing steroid-responsive disease. Long-term respiratory rehabilitation may be relevant when dyspnea or exercise limitation persists after the acute episode (9, 54). Antifibrotic strategies remain investigational in CIP and should be considered experimental rather than routine (36, 68).

### 8.6 ICI rechallenge

ICI rechallenge after CIP should be considered an individualized and carefully selected option rather than a routine consequence of clinical recovery. The decision should take into account the severity of the initial CIP, the degree of clinical and radiographic resolution, residual oxygen or pulmonary function impairment, corticosteroid requirements, prior steroid-refractory or steroid-relapsing behavior, the availability of alternative anticancer therapies, and the expected oncological benefit. Rechallenge may be considered in selected patients with complete or near-complete clinical and radiographic recovery, low-risk initial disease, and a favorable oncological rationale. In contrast, patients with severe, recurrent, steroid-refractory, steroid-dependent, or incompletely resolved CIP generally require a more conservative approach.

The safety of ICI rechallenge after CIP has not been firmly established. Current evidence is derived mainly from retrospective cohorts and selected clinical series, with potential selection bias and heterogeneous definitions of recovery, recurrence, and rechallenge eligibility (10, 23). Accordingly, the absence of active symptoms alone should not be interpreted as evidence that rechallenge is safe. When rechallenge is considered, it should follow multidisciplinary review and shared decision-making, with explicit discussion of recurrent pneumonitis risk, the availability of alternative anticancer therapies, and the expected oncological benefit (19, 36, 37). Monitoring should be more intensive than routine follow-up, with close attention to recurrent symptoms, oxygenation, pulmonary function, and imaging changes. Rechallenge should be discontinued or reconsidered if clinically meaningful pulmonary deterioration occurs (37).

## 9 Discussion

### 9.1 Clinical implications and current controversies

Taken together, phenotype-guided therapy emphasizes proportionality. Mild disease may be observed, moderate disease usually warrants corticosteroids, severe disease requires urgent inpatient management, steroid-refractory disease may justify selected second-line immunomodulation, chronic disease needs longer-term pulmonary follow-up, and rechallenge should be reserved for carefully chosen cases. This framework is deliberately practical: it does not replace existing guidelines, but it helps explain why treatment intensity should be matched to phenotype rather than grade alone (18, 22, 35).

The balance between pulmonary control and oncologic efficacy is also uncertain (10). More intensive immunosuppression may improve lung outcomes in selected patients, but it could also increase infection risk and potentially affect antitumor immunity. The long-term pulmonary consequences of different steroid tapering strategies and second-line agents are likewise not well defined (16, 54). Rechallenge data remain limited, and current practice is guided more by clinical judgment than by robust comparative evidence (36, 37).

Taken together, these limitations mean that phenotype-guided management should be viewed as a practical framework rather than a validated algorithm. Its value is that it helps organize imperfect evidence into a clinically usable structure. The framework is strongest when it promotes more careful reasoning, better multidisciplinary communication, and more explicit recognition of uncertainty (19, 21, 54). It is weakest if it is treated as a substitute for prospective validation, standardized phenotyping, or biomarker development (22, 35). These gaps lead directly to the future research priorities discussed in the next section.

### 9.2 Evidence gaps and limitations

Despite rapid progress in understanding CIP, the most clinically relevant evidence gaps remain biomarker validation and immunomodulatory treatment evidence. The most important limitation is that much of the available literature is observational, retrospective, and derived from single-center experience (11, 33). This makes it difficult to separate true biological effects from differences in case definition, surveillance intensity, treatment threshold, and referral patterns. As a result, apparent incidence rates, risk factors, and treatment responses are often not directly comparable across studies (39, 40, 48).

Current limitations are most relevant in two areas: biomarker validation and immunomodulatory treatment evidence. No single biomarker is yet sufficient to diagnose CIP on its own, and most blood-based, BALF-based, and tissue-based signals remain complementary rather than definitive. Likewise, evidence for second-line immunomodulatory therapy is still limited, with most data derived from retrospective series, small cohorts, or case-based experience. These gaps underscore the need for prospective phenotyping, clinically accessible biomarkers, and better-defined treatment pathways.

#### 9.2.1 Methodological limitations

CIP is not defined uniformly across all reports, and this alone creates substantial heterogeneity. Some studies rely on clinical judgment, some on imaging criteria, and others on stricter exclusion of infection or alternative diagnoses. Different grading systems, different imaging thresholds, and different approaches to bronchoscopy or microbiological testing further complicate comparison (24, 55). BALF and lung tissue studies are especially vulnerable to sampling bias, because the patients who undergo invasive testing are often those with more severe, atypical, or diagnostically uncertain disease (10, 29, 67).

The time course of CIP is another source of methodological difficulty. A patient classified at one time point may later evolve into a different phenotype, particularly if corticosteroids are started early or if tapering triggers relapse. Many studies capture only the initial presentation, which can underrepresent chronic, relapsing, or steroid-dependent disease (16, 17). This is one reason why longitudinal phenotyping is more informative than single time-point classification (18, 22, 54).

#### 9.2.2 Limitations of current biomarkers

At present, no biomarker has been validated to diagnose CIP on its own. Blood-based markers such as neutrophil-to-lymphocyte ratio, cytokines, albumin, or inflammatory indices may help with risk estimation, but their specificity is limited. BALF findings may be biologically closer to the lung lesion, yet they are invasive, not standardized, and often unavailable before treatment begins (56, 60, 67). Single-cell and tissue profiling are informative but remain research tools rather than routine clinical assays (29, 30).

The main challenge is translation. Many promising immune signatures are difficult to reproduce across platforms, cohorts, or treatment settings. Even when a biomarker appears associated with CIP, it may not distinguish between pre-existing lung disease, infection, radiation injury, or another pulmonary adverse event (28, 42). For this reason, current biomarkers are best viewed as complementary signals rather than diagnostic endpoints (35, 44, 60).

#### 9.2.3 Limitations of immunomodulatory treatment evidence

Evidence for second-line immunomodulatory therapy remains particularly weak. Outside corticosteroids, most options have been supported by retrospective series, case reports, small cohorts, or extrapolation from other immune-mediated toxicities. This makes it difficult to determine which patients truly benefit from treatment escalation, which agent is optimal for a given phenotype, and when immunosuppression becomes more harmful than helpful (17, 35, 68). These gaps define the key priorities for future research discussed below.

### 9.3 Future research directions

The most useful future studies in CIP will be those that move the field from descriptive observation toward clinically actionable stratification. The next phase of research should not simply generate more biomarkers or more immune signatures; it should determine which measurements genuinely improve diagnosis, risk prediction, treatment selection, and safe immune checkpoint inhibitor rechallenge. To achieve that goal, future work will need to be prospective, longitudinal, and better aligned with the clinical phenotypes that matter at the bedside (18, 22, 23).

#### 9.3.1 Standardized CIP phenotyping

A priority is the development of a standardized phenotyping system that combines clinical, radiographic, laboratory, bronchoalveolar lavage, and tissue data. Such a system should define how to classify asymptomatic, symptomatic, hypoxemic, diffuse injury, relapsing, and chronic phenotypes in a reproducible way. It should also specify how to incorporate time course, steroid response, and competing diagnoses. Without standardized phenotyping, it will remain difficult to compare cohorts, validate biomarkers, or test phenotype-guided interventions across centers (22, 54).

A useful framework would distinguish between phenotypes that are stable at presentation and phenotypes that evolve over time. This would allow future studies to move beyond one-time grading and instead capture the dynamic biology of CIP (22, 30).Standardized phenotyping would also make it easier to link imaging, BALF, and peripheral blood findings to clinically meaningful outcomes (46, 67).

#### 9.3.2 Clinically accessible biomarkers

The field needs biomarkers that are practical, inexpensive, and available early enough to influence decision-making. Candidate markers include ANC, neutrophil-to-lymphocyte ratio, IL-17A, IL-6, GSDME-related signals, CD8+ tissue-resident memory-related markers, Th17.1-related markers, and imaging-derived features (29, 73). None of these is ready to serve as a standalone diagnostic test. Their value will depend on whether combinations of markers outperform clinical judgment alone in prediction of severity, steroid responsiveness, or recurrence (33, 47, 74).

Future biomarker studies should therefore focus on multi-parameter models rather than single analytes. The most promising approach may be a layered model that integrates blood-based markers, radiographic features, and selectively obtained BALF data. Such models should be tested for calibration, transportability, and clinical utility rather than only for statistical association (22, 33, 74).

#### 9.3.3 Prediction of steroid-refractory CIP

Predicting steroid-refractory disease is one of the most important unmet needs (17). The patients most likely to benefit from early recognition are also the patients most likely to deteriorate quickly if treatment is delayed (33). Future studies should examine whether baseline interstitial lung disease, CT disease burden, inflammatory indices, hypoxemia, procalcitonin, albumin, and early corticosteroid response can be combined into a reliable risk model. Immune-cell signatures may add biological depth, but they should be evaluated in parallel with simpler clinical markers to determine whether they provide actionable incremental value (29, 30, 56).

Ideally, risk prediction should occur early enough to influence whether second-line immunomodulation is started promptly rather than after clear progression has already occurred (17, 54). This would require prospective studies with prespecified time points, treatment pathways, and outcomes (35).Steroid-refractory CIP is too clinically consequential to remain an after-the-fact descriptive category (17, 33).

#### 9.3.4 Phenotype-guided immunomodulation

Future studies should also clarify which immune phenotypes are most likely to benefit from specific immunomodulatory strategies. It is biologically plausible that IL-6, TNF, IL-17, or IL-23 blockade may not be interchangeable across patients with different inflammatory programs. The challenge is to determine whether such pathway-based treatment actually improves pulmonary outcomes without unacceptable infection risk or compromise of antitumor efficacy (7, 54). This will require careful patient selection and, ideally, prospective comparative studies rather than isolated case reports (68, 72, 75).

Another priority is safer and more individualized ICI rechallenge. Future research should define which patients can be rechallenged after complete or partial resolution, how much residual radiographic abnormality can be tolerated, and what monitoring strategy best detects recurrence early (16, 37). Rechallenge is currently guided largely by clinical judgment, but its safety would be easier to manage if the pre-rechallenge phenotype could be more precisely defined (23, 68).

#### 9.3.5 Integrating research with clinical decision-making

The broader goal of future work should be to link biology with action. The most valuable studies will be those that answer practical questions: Who needs early bronchoscopy? Who can be observed safely? Who is likely to respond to corticosteroids alone? Who needs early escalation? Who can be rechallenged, and under what conditions? If future research can answer these questions in a reproducible way, CIP management will move from reactive toxicity treatment toward genuinely phenotype-guided care (18, 35).

In that sense, the field does not simply need more data. It needs better-aligned data: data collected with standardized phenotypes, analyzed in relation to treatment response, and interpreted in a way that improves bedside decisions (22, 23, 54). That is the standard against which future CIP studies should be judged (35).

## 10 Conclusions

CIP is not a single clinical entity, but a dynamic and heterogeneous immune-mediated pulmonary syndrome with variable severity, radiographic appearance, inflammatory activity, and corticosteroid responsiveness. This heterogeneity helps explain why CTCAE grade alone often fails to capture the full biological and therapeutic complexity of the disease at the bedside (18, 19, 23). A limited radiographic burden with rapid steroid response, for example, should not be interpreted in the same way as diffuse alveolar injury with early steroid resistance or relapse during tapering (15–17).

The framework proposed in this review integrates four dimensions: clinical severity, radiographic phenotype, inflammatory and immune phenotype, and corticosteroid responsiveness. In practice, this approach may support earlier recognition of high-risk disease, improve diagnostic confidence when alternative causes remain plausible, and help guide escalation, tapering, and follow-up decisions more precisely (28, 33). It may also provide a more rational basis for considering immune checkpoint inhibitor rechallenge after recovery (36, 37).

At present, this framework should be viewed as a clinically grounded heuristic rather than a validated classification system (22, 35). Prospective multicenter studies with standardized phenotyping and outcome definitions are still needed to determine whether phenotype-guided management can improve both pulmonary safety and oncologic continuity (22, 23, 54).
